# Host immunoglobulin G selectively identifies pathobionts in pediatric inflammatory bowel diseases

**DOI:** 10.1186/s40168-018-0604-3

**Published:** 2019-01-03

**Authors:** Heather Armstrong, Misagh Alipour, Rosica Valcheva, Michael Bording-Jorgensen, Juan Jovel, Deenaz Zaidi, Prachi Shah, Yuefei Lou, Cory Ebeling, Andrew L. Mason, Dawson Lafleur, Jeremy Jerasi, Gane K.-S. Wong, Karen Madsen, Matthew W. Carroll, Hien Q. Huynh, Levinus A. Dieleman, Eytan Wine

**Affiliations:** 1 CEGIIR, University of Alberta, Edmonton, AB T6G 2X8 Canada; 2 Department of Pediatrics, University of Alberta, Edmonton Clinic Health Academy, Room 4-577, 11405 87th Ave, Edmonton, AB T6G 1C9 Canada; 3 Department of Physiology, University of Alberta, Edmonton, AB T6G 1C9 Canada; 4 Department of Medicine, University of Alberta, Edmonton, AB T6G 2G3 Canada; 5 Department of Medical Microbiology and Immunology, University of Alberta, Edmonton, AB T6G 2G3 Canada; 6 Department of Biological Sciences, University of Alberta, Edmonton, AB T6G 2G3 Canada

**Keywords:** Crohn disease, Immunoglobulins, Microbiota, Ulcerative colitis, Dysbiosis

## Abstract

**Background:**

Inflammatory bowel diseases (IBD) are a group of complex and multifactorial disorders with unknown etiology. Chronic intestinal inflammation develops against resident intestinal bacteria in genetically susceptible hosts. We hypothesized that host intestinal immunoglobulin (Ig) G can be used to identify bacteria involved in IBD pathogenesis.

**Results:**

IgG-bound and -unbound microorganisms were collected from 32 pediatric terminal ileum aspirate washes during colonoscopy [non-IBD (*n* = 10), Crohn disease (*n* = 15), and ulcerative colitis (*n* = 7)], and composition was assessed using the Illumina MiSeq platform. In vitro analysis of invasive capacity was evaluated by fluorescence in situ hybridization and gentamicin invasion assay; immune activation was measured by qPCR. Despite considerable inter-individual variations, IgG binding favored specific and unique mucosa-associated species in pediatric IBD patients. *Burkholderia cepacia*, *Flavonifractor plautii*, and *Rumminococcus* sp*.* demonstrated increased IgG binding, while *Pseudomonas* ST29 demonstrated reduced IgG binding, in IBD. In vitro validation confirmed that *B. cepacia*, *F. plautii*, and *Rumminococcus* display invasive potential while *Pseudomonas protogens* did not.

**Conclusion:**

Using IgG as a marker of pathobionts in larger patient cohorts to identify microbes and elucidate their role in IBD pathogenesis will potentially underpin new strategies to facilitate development of novel, targeted diagnostic, and therapeutic approaches. Interestingly, this method can be used beyond the scope of this manuscript to evaluate altered gut pathobionts in a number of diseases associated with altered microbiota including arthritis, obesity, diabetes mellitus, alcoholic liver disease, cirrhosis, metabolic syndrome, and carcinomas.

**Electronic supplementary material:**

The online version of this article (10.1186/s40168-018-0604-3) contains supplementary material, which is available to authorized users.

## Background

Inflammatory bowel diseases (IBD) include the chronic and severely debilitating, immune-mediated disorders known as Crohn disease (CD) and ulcerative colitis (UC) [1, 2]. North America and Northern Europe demonstrates some of the highest prevalence rates of IBD in the world affecting approximately 1 in 150 Canadians, and incidence rates are rising, especially in children, who make up 25% of newly diagnosed cases each year [3–6]. The etiology of IBD remains poorly understood; however, it is recognized to be multifactorial with both hereditary and environmental factors, such as urban lifestyle, dietary factors, heightened hygiene, and the gut microbiota involved in disease development [1, 2, 7–13]. The intestinal microbiome is critical to maintain human health and is involved in mediating key functions of metabolism and the immune system [8, 14]. Altered gut microbiota has been associated with a number of diseases including arthritis, obesity, diabetes mellitus, alcoholic liver disease, cirrhosis, metabolic syndrome, carcinomas, and IBD [15–23]. Microbial diversity is commonly reduced in IBD, and this can be used as a predictive marker for failure to respond to therapy in severe pediatric UC [24]. Dysbiosis, or compositional changes in the intestinal microbiota, is a hallmark of IBD, along with defects in the gastrointestinal barrier and a loss of immune tolerance [1, 2, 12–14].

Recognition of enteric microbes by the intestinal immune system results in the production of immunoglobulin (Ig) A and IgG, predominant antibody isotypes found at the intestinal mucosal surface [25, 26]. These antibodies provide protection against infection by binding to and coating pathogens within the intestinal lumen. Studies completed by our group [27], and others [28, 29] suggest that coating of intestinal bacteria with high-affinity IgA can be used to identify pathogenic strains involved in the development of IBD. A recent study by D’Auria et al. supported our hypothesis as they reported a difference in taxa bound by IgA compared to total bacteria in healthy subjects [30]. More recently, Palm et al. elucidated a role for IgA-bound bacteria isolated from the stool of IBD patients in inducing colitis in nude mice [28].

While previous studies focused on IgA as a marker of pathobionts support our hypothesis in general, focusing on IgG-bound bacteria may provide even more clinically relevant results as the majority of secretory IgA within the intestine binds bacteria non-selectively, resulting in the identification of commensal bacteria [31]. In contrast, IgG in the lumen has been shown to be elevated in IBD patients [32–34] and IgG+ intestinal plasmablasts are greater than 3-fold more reactive to specific pathobionts than IgA+ plasmablasts [35, 36], demonstrating IgG’s pathogen-binding specificity. Furthermore, while IgA remains the predominantly produced immunoglobulin in the intestinal tract of healthy patients, studies have demonstrated a predominance of IgG in IBD patients [37]. Therefore, we have focused on IgG-bound bacteria.

The specific responses of the adaptive immune system, which is more involved in IgG vs IgA production, towards these luminal antigens are altered in IBD patients [38, 39]. Our group has further demonstrated that the mucosal layer structure and mucosal microbial composition are altered in unaffected ileal tissues from pediatric UC patients [27], suggesting a link for microbial involvement in UC pathogenesis, and not just a change secondary to inflammation. For this reason, we have examined only unaffected regions of the terminal ileum (TI), determined by histology.

There is relatively little direct interaction between microorganisms found in the intestinal lumen and the underlying epithelium, which are separated by the mucus layer [40, 41]. While many microorganisms have been linked to disease, those associated with the mucosa are more likely to play a role in IBD pathogenesis than those within the lumen due to their proximity to the intestinal epithelium [40, 41]. These microorganisms, therefore, have the potential to invoke the characteristic inflammatory symptoms of these diseases.

Based on these data, we hypothesized that bacteria more highly bound by IgG would display greater virulence than unbound strains and that identification of these invasive, pathobiont strains (defined as pathogenic only under specific conditions [42]) will assist in better understanding the mechanisms of intestinal immune activation and aberrant immune reactivity to microbes in IBD pathogenesis. This *proof-of-principle study* reveals that increased IgG binding to microorganisms collected from intestinal washes of the mucosal epithelium of pediatric IBD patients allows for selective identification of specific microorganisms that display pathobiont properties and, therefore, may be involved in driving or exacerbating IBD.

## Results

### Optimization of bacterial cell sorting in mixed culture

As IgG antibodies provide protection to the intestinal mucosa by binding and coating pathogens, we hypothesize that coating of intestinal bacteria with high-affinity IgG can be used to identify pathogenic strains that have previously stimulated a humoral response, and are therefore more likely to be invasive/pathobionts, involved in the development of IBD. To first evaluate the sensitivity of fluorescence-activated cell sorting (FACS) in recognizing and isolating bacteria in vitro, lipopolysaccharide (LPS) surface staining of *Escherichia coli* HB101 was determined. This demonstrated that indeed, 53% of *E. coli* were positively stained compared to 2% in the isotype control sample (Additional file 1: Figure S1A). To further demonstrate the ability of FACS to adequately sort bacteria, *E. coli* HB101 and *Lactobacillus reuteri* were co-cultured 1:1 (Additional file 1: Figure S1B) and *E. coli* were stained using an anti *E. coli* O+K antibody, then separated by FACS gating. Purity and viability were determined by growth patterns on specific agar plates (confirmed and quantified by qPCR; Additional file 1: Figure S1C), showing extremely high purity of *E. coli* (grown on MacConkey and MRS agar plates; 95–98%) and excellent viability after FACS sorting. These results demonstrate our ability to isolate and culture antibody-bound bacteria from a mixed population using FACS, as also demonstrated by others [28].

### Isolation of IgG-coated bacteria from pediatric IBD intestinal washes

**Fig. 1:**
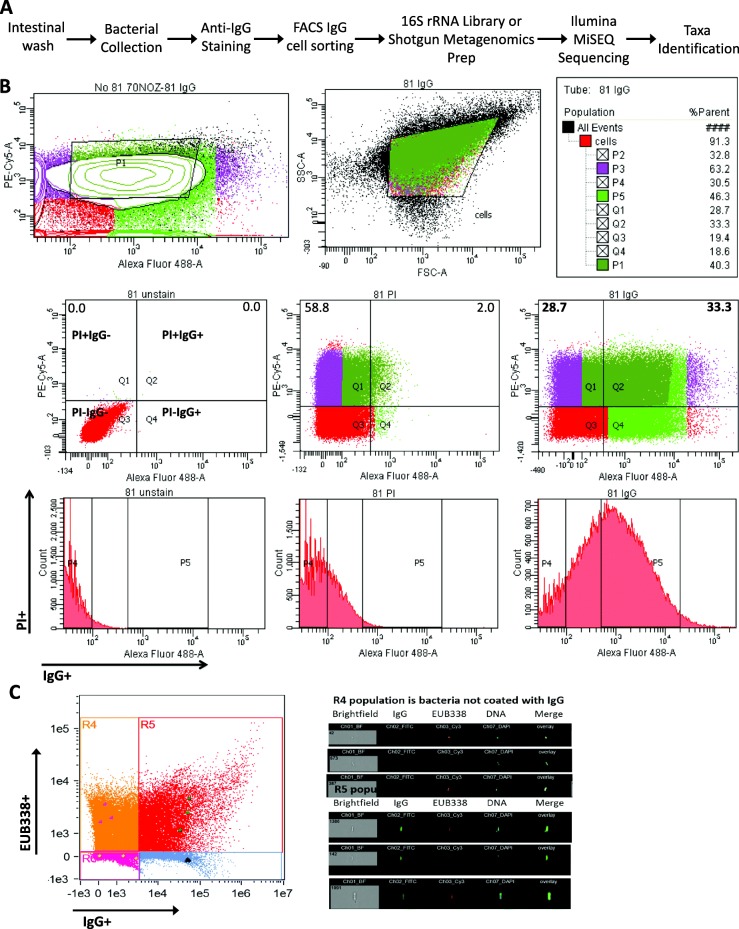
IgG cell sorting and 16S rRNA gene sequencing identify microbes collected from pediatric IBD patient ileal wishes. **a** Sequence of procedures for the identification of IgG-bound pathobiont microbes. **b** Representative of cell sorting of patient sample #81 by BD FACSAria III with a shift in population of IgG+ bacteria (33.3%). The Q1 and Q2 quadrants comprise PI positive/IgG negative (IgG−) and PI positive/IgG positive (IgG+), respectively. **c** Representative of image flow cytometry with shift in population of IgG+ bacteria. The Amnis ImagestreamX mkII was used for acquisition of image cytometry, performed at × 60 magnification. Gating compensation was performed using individual stained (DAPI, EUB338, or anti-IgG) sample controls. Analysis was performed using IDEAS Software version 6.1

To characterize the composition of the intestinal microbiota of pediatric IBD and non-IBD control patients, luminal wash samples were processed through a series of steps as displayed in Fig. 1a. We utilized the binding of microbes by patient-derived IgG, which occurs naturally within the gut of patients to then separate these IgG-bound bacteria using FACS. Intestinal wash samples were collected from pediatric non-IBD (*n* = 10; mean age 13.4 years), CD (*n* = 15; mean age 12.6 years), and UC (*n* = 7; mean age 12.7 years) patients at the University of Alberta Stollery Children’s Hospital in Edmonton, Canada (see patient characteristic summary in Additional file 1: Table S1; further details on individual patients are highlighted in Additional file 1: Table S2). IgG-coated microbes were distinguished from non-IgG-coated microbes by FACS using an anti-human IgG antibody for visualization (Fig. 1b; Additional file 1: Table S3). A minimum of 6 million events were collected per samples (IgG+ and IgG−). All samples were first fixed using paraformaldehyde to “kill” and permeabilize the microbial cells and to secure that IgG will remain bound to bacteria. Propidium iodide (PI) was then utilized to stain these fixed microbes to separate out all food particulate, mucous, and non-microbes from the intestinal wash supernatants (only intact, fixed microbes would retain the PI; all PI-negative particles would not be bacteria). This allowed us to appropriately examine IgG binding to microbes rather than background noise from these samples. Bacteria positive for both PI (stains DNA to identify bacterial cells) and IgG were labeled IgG+, and those positive for PI but not IgG were designated IgG−. An average of 33.7% (non-IBD), 29.7% (CD), and 34.2% (UC) of each sample was sorted as the IgG+ fraction, while 34.8% (non-IBD), 25.6% (CD), and 35.8% (UC) was sorted to IgG− (*p* > 0.05 for all). Roughly 10^7^ CFU/mL bacterial cells were collected per sample in order to obtain enough DNA for sequencing.

To confirm the ability of FACS to differentiate IgG+ from IgG− bacteria, as well as to validate the specificity and sensitivity of FACS to detect bacteria only, and no other particles, the sorted samples were assessed in parallel using image cytometry (Fig. 1c). Bacteria were stained with EUB338 eukaryote stain and IgG antibody, and coinciding images demonstrate accurate cell sorting of IgG− and IgG+ bacteria using FACS, allowing for comparative sequencing.

### Microbial composition of ileal isolates from pediatric IBD patients using 16S rRNA gene libraries

**Fig. 2:**
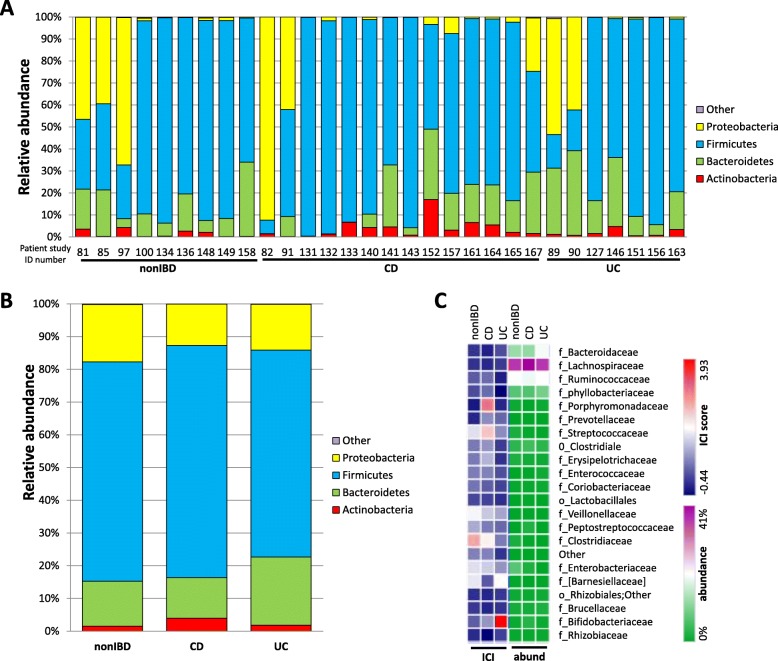
Ilumina MiSeq sequencing of 16S rRNA gene library demonstrates **a** the relative abundance of phyla per patient and **b** average among patients in non-IBD (*n* = 9), CD (*n* = 14), and UC (*n* = 7) pediatric patient samples. **c** Heatmap depicting the mean IgG Coating Index (ICI) scores and average total (IgG+ and IgG−) relative abundance (abund) of bacterial family level taxa for non-IBD, CD, and UC sample sets. ICI scores are depicted on a logarithmic scale; taxa are presented at the closest family/order

Mucosa-associated bacteria were collected from the TI during endoscopy by washing the surface with normal saline (0.9%) and aspirating the content. Several studies have shown the utility of intestinal washes in capturing microbes in close contact to the mucosa and that these are very different from microbes found in the stool [43, 44]. This is achieved by direct disruption and collection of the mucus layer during endoscopy. Although we did not compare the microbes identified in our study to the luminal ones, the lumen is usually dry after bowel prep and is less likely to contain sufficient bacteria for comparison. Bacteria were then fixed and spun down, and DNA extracted, as described in the “Methods” section. The composition of ileal microbiota of pediatric patients was then examined by Illumina MiSeq sequencing on a total of 15 CD and 7 UC patients and 10 non-IBD control subjects (Fig. 1a). While all patient samples were sequenced on the Illumina MiSeq platform, two libraries were constructed—one for 16S rRNA gene analysis and the other for metagenomics. For results referring to 16S rRNA gene, the 16S rRNA bacterial gene was PCR amplified to identify bacteria in non-IBD (*n* = 9), CD (*n* = 14), and UC (*n* = 7) pediatric patient intestinal washes at the phylum level (Fig. 2a, b) and the family level (Fig. 2c). Relative abundance of all major phyla identified by Illumina MiSeq was calculated for the 16S rRNA gene (Fig. 2a) libraries. Results from the 16S rRNA gene library displayed no gross statistical difference in relative abundance at the phylum level with predominance in Firmicutes, Proteobacteria, and Bacteroidetes in all patient cohorts (Fig. 2a, b). Only microbes with abundance greater than 1% were included for analysis.

In order to compare binding levels of IgG between taxa of non-IBD, CD, and UC patients, we calculated the IgG Coating Index (ICI) score for each bacterial family (ICI = relative abundance [IgG+]/relative abundance [IgG−] [28]). Although ANOVA demonstrated no statistically significant changes in ICI, the median ICI scores at the family level indicated a trend towards IgG binding favoring *Clostridiaceae* in non-IBD, *Porphyromonadaceae* in CD, and *Bifidobacteriaceae* in UC (Fig. 2c). These changes in ICI were not the result of altered total abundances as abundance remained relatively constant between non-IBD, CD, and UC cohorts (Fig. 2c; right side of heatmap). This highlights that ICI is not just a reflection of altered abundance but rather represents a separate process. While family level resolution identified using the 16S rRNA gene library samples was not sufficient to show a significant change in ICI, these results did establish the ability of IgG staining to identify favored genera at the individual patient level.

### Altered relative abundance of bacterial species using shotgun metagenomics

**Fig. 3:**
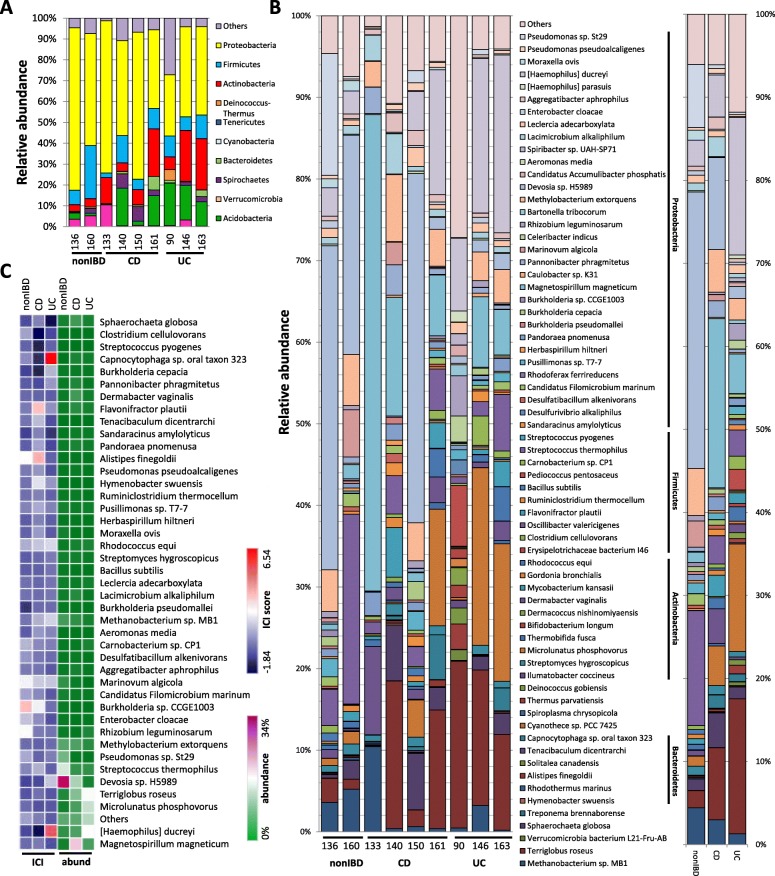
Ilumina MiSeq sequencing of shotgun metagenomic libraries demonstrates changes in the relative abundance of **a** phyla and **b** species taxa in non-IBD (*n* = 2), CD (*n* = 4), and UC (*n* = 3) pediatric patient samples. The panel on the right indicates the mean species relative abundance per group. **c** Heatmap depicting the mean IgG Coating Index (ICI) scores and average total (IgG+ and IgG−) relative abundance (abund) of bacterial species for non-IBD, CD, and UC sample sets. ICI scores are depicted on a logarithmic scale

To gain more in-depth, accurate, and detailed sequencing data (as 16S rRNA gene analysis was not sufficient for species level analysis), a small number of patient samples were examined by shotgun metagenomics (due to more stringent requirements, several samples used for 16S rRNA analysis did not meet quality criteria for metagenomics). For the shotgun metagenomics sample set, unrestricted sequencing was performed (i.e., included all parts of the bacterial genomes, which were initially fragmented enzymatically) to identify bacteria in non-IBD (*n* = 2), CD (*n* = 4), and UC (*n* = 3) pediatric patient TI washes at the species level. No significant changes were found in the inter-patient variability of bacterial taxon relative abundance at the phylum level (Fig. 3a) with predominantly Proteobacteria, Firmicutes, and Actinobacteria, although changes were more noticeable at the species level (Fig. 3b). Notable changes in relative abundance are highlighted in Table 1. While patterns of relative abundance, detailed in Table 1, were indicative of changes in some patients, the only change in relative abundance that demonstrated statistical significance among all patients was a lowered relative abundance of *Moraxella ovis* (*Proteobacteria*; 1.21% non-IBD, 0.20% CD, 0.10% UC) in non-IBD compared to both CD and UC (*p* < 0.001).

**Table 1:** Bacterial relative abundance and ICI score based on shotgun metagenomics library sequencing data (***p < 0.001)

Phyla	Species	% non-IBD	% CD	% UC	High abundance in
Euryarchaeota	*Methanobacterium* sp. *MB1*	4.42	2.97	1.29	non-IBD
Firmicutes	*Streptococcus thermophiles*	13.86	3.41	3.17	non-IBD
Proteobacteria	*Devosia* sp. *H5989*	33.29	11.08	1.45	non-IBD
Proteobacteria	*Marinovum algicola*	3.14	0.74	0.02	non-IBD
Proteobacteria	*Pseudomonas* sp. *St29*	7.62	0.44	0.29	non-IBD
Acidobacteria	*Terriglobus roseus*	2.08	8.68	16.27	IBD
Actinobacteria	*Microlunatus phosphovorus*	1.2	4.77	12.98	IBD
Actinobacteria	*Dermabacter vaginalis*	0.66	4.19	1.05	IBD
Bacteroidetes	*Capnocytophaga* sp*. oral taxon 323*	0.06	1.6	0.93	IBD
Proteobacteria	*Magnetospirillum magneticum*	1.23	20.37	4.77	IBD
Proteobacteria	*Haemophilus] ducreyi*	3.14	5.05	16.55	IBD
Spirochaetes	*Sphaerochaeta globosa*	1.37	4.17	1.42	IBD
Phyla	Species	ICI non-IBD	ICI CD	ICI UC	High IgG bound in
Firmicutes	*Clostridium cellulovorans*	2	0.337209	0.75	non-IBD
Proteobacteria	*Moraxella ovis*	1.21	0.2	0.1	non-IBD ***
Proteobacteria	*Burkholderia* sp. *CCGE1003*	11.572222	4.75	1.142857	non-IBD
Proteobacteria	*Rhizobium leguminosarum*	5.371875	1.307403	1.210941	non-IBD
Bacteroidetes	*Hymenobacter swuensis*	1.1666667	3.979167	1.75	CD
Bacteroidetes	*Alistipes finegoldii*	5.625	13.28889	0.997768	CD
Bacteroidetes	*Tenacibaculum dicentrarchi*	1.2	2.604167	0.805195	CD
Firmicutes	*Flavonifractor plautii*	1.2913257	11.15111	1.643084	CD
Proteobacteria	*Sandaracinus amylolyticus*	0.6924342	1.424057	0.608021	CD
Proteobacteria	*Pandoraea pnomenusa*	0.9506579	2.007981	0.736745	CD
Bacteroidetes	*Capnocytophaga* sp. *oral taxon 323*	1.5625	0.57436	93	UC
Proteobacteria	*Burkholderia cepacia*	0.6970721	0.515473	2.468487	UC
Proteobacteria	*Pannonibacter phragmitetus*	0.912963	0.769207	1.817716	UC
Proteobacteria	*Devosia* sp*. H5989*	0.7209405	0.761257	2.919889	UC
Proteobacteria	*[Haemophilus] ducreyi*	0.7542442	0.385538	35.89216	UC
Archaea	*Methanobacterium* sp. *MB1*	1.2109844	2.36367	3.378841	CD and UC
Proteobacteria	*Burkholderia pseudomallei*	0.6134085	1.091992	1.097222	CD and UC

### Preferential binding of IgG differs among non-IBD, CD, and UC patients

**Fig. 4:**
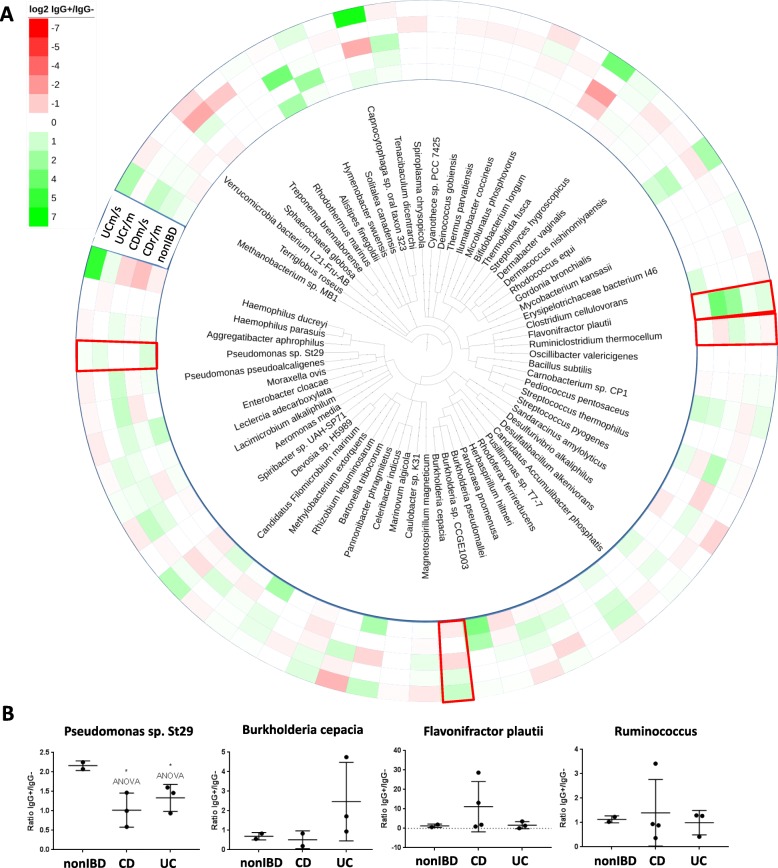
Ratio of IgG+/IgG− binding (ICI) in shotgun metagenomic library patient samples. **a** The ICI for each species identified was calculated for non-IBD (*n* = 2), remission/mild CD (CDr/m; *n* = 2), moderate/severe CD (CDm/s; *n* = 2), remission/mild UC (UCr/m; *n* = 1), and moderate/severe UC (UCm/s; *n* = 2) and is expressed in a cladogram using log_2_-based heatmap coding. Red boxes highlight species selected for in vitro examination. **b** Ratio of IgG binding (ICI) for four of the bacterial species of interest in which each dot represents an individual patient, offering further validation

While there were limited changes in overall ICI between the groups, several bacterial taxa within each of the patient groups showed preference for binding IgG (Fig. 3c, Fig. 4b, Additional file 1: Figures S2, S3). Based on results from the shotgun sequencing libraries, IgG bound preferentially to *Candidatus Filomicrobium marinum*, *Carnobacterium* sp. CP1, *Caulobacter* sp. K31, *Desulfatibacillum alkenivorans*, *Enterobacter cloacae*, *Burkholderia* sp. CCGE1003, *Pseudomonas* sp. St29, and *Rhizobium leguminosarum* in non-IBD (Additional file 1: Figure S2A). A number of microbes did not display any preferential IgG binding between non-IBD and IBD groups (Additional file 1: Figure S2B). *Aeromonas media*, *Burkholderia pseudomallei*, and *Streptomyces hygroscopicus* display increased ICI in both CD and UC patients (Additional file 1: Figure S2C). *Celeribacter indicus*, *Herbaspirillum hitneri*, *Hymenobacter swuensis*, *Alistipes finegoldii*, *Lacimicrobium alkaliphilum*, *Moraxella ovis*, *Ruminiclostridium thermocellum*, *Spharochaeta globose*, *Terriglobus roseus*, *Tenacibaculum dicentrarchi*, *Flavonifractor plautii*, *Sandaracinus amylolyticus*, and *Pandoraea pnomenusa* in CD (Additional file 1: Figure S3A). *Capnocytophaga* sp*.* oral taxon 323, *Burkholderia cepacia*, *Pannonibacter phragmitetus*, *Devosia* sp*.* H5989, *Rhodococcus equi*, *Thermobifida fusca*, *[Haemophilus] ducreyi*, and *Methanobacterium* sp*.* MB1 in UC (Additional file 1: Figure S3B). Again, these changes in ICI were not associated with any changes in species abundance (Fig. 3c). Given the small numbers, many of these differences did not reach statistical significance by ANOVA with the exception of a significant reduction in ICI to *Pseudomonas* sp. St29 (*p* < 0.05) in IBD compared to non-IBD. Still, these findings do suggest that some bacterial species preferentially bind to IgG in a disease-specific manner.

To identify potential microbes associated with CD or UC pathogenesis, as indicated by altered ICI, we examined changes in ileal mucosa-associated microbiota composition at different severity levels of the disease from remission/mild to moderate/severe inflammation, compared to non-IBD (Fig. 4a). Red boxes highlight the microbes selected for in vitro studies. While the number of patients per group here is too low to identify significant changes, results suggest a similar pattern to the changes described between non-IBD and total CD or UC and a possible “dose effect” (Fig. 3c, Additional file 1: Figures S2, S3).

### The pathogenic potential of identified species offers proof of principle for IgG-binding pathobionts

A select series of microbes identified as having increased or decreased binding to IgG within the pediatric TI were examined in vitro to determine their invasive potential, as a surrogate for virulence and likelihood of inducing an immune response. The Proteobacterium *B. cepacia* demonstrated a 2.5-fold and 4.1-fold increase in the ICI in remission/mild (r/m) or moderate/severe (m/s) UC, respectively, compared to non-IBD (Fig. 4b). *B. cepacia* has previously been identified in the ileum of IBD cohorts [45] and has been reported to display pro-inflammatory effects and invasive potential in an in vitro model, supporting its pathobiont potential [46, 47]; however, it is not typically recognized as an intestinal pathogen or pathobiont. To further substantiate these results, we also examined the Firmicute species, *F. plautii* (increased ICI by 11.8-fold r/m; 5.5-fold m/s in CD), and *Ruminococcus* sp*.* (increased ICI by 2-fold m/s in CD; Fig. 4b), which we were able to isolate live from pediatric IBD patients. Both *F. plautii* and *Ruminococcus* have been mentioned in previous studies aimed at identifying gut microbes [48], while only *Ruminococcus* has been previously associated with human disease, including arthritic inflammation [49]. Interestingly, the Proteobacteria, *Pseudomonas* sp. ST29, previously studied in the rhizosphere of rice and potato [50], displayed a statistically significant decrease in ICI (2.1-fold in CD; 1.5-fold in UC). As we were unable to isolate live *Pseudomonas* sp. ST29, we purchased the related *Pseudomonas protogens* Pf-5 strain from ATCC, which displays 96% sequence homology as it was the closest isolate available. *Pseudomonas protogens* Pf-5 has been shown to display similar characteristics to St29 [51].

**Fig. 5:**
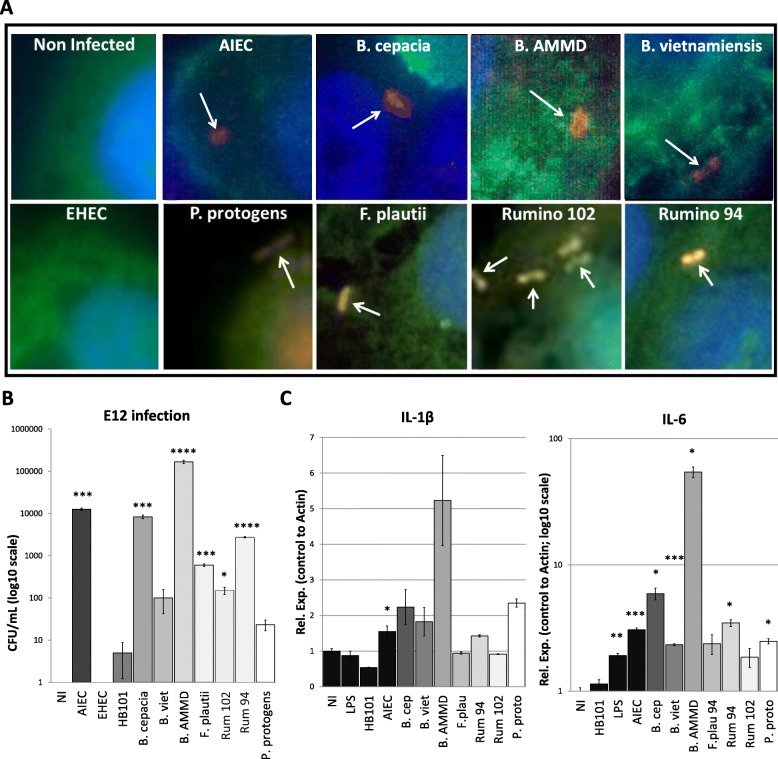
IgG-bound bacteria from IBD patients demonstrate invasive characteristics in human intestinal cell culture. Invasive capacity of *Burkholderia cepacia*, *Burkholderia vietnamiensis*, *Burkholderia ambifaria*, *Flavonifractor plautii*, *Ruminococcus* sp*.* patient 94 (CD), *Ruminococcus* sp*.* patient 102 (non-IBD), and *Pseudomonas protogens* in the HT29-MTX-E12 cell line was examined by **a** fluorescence in situ hybridization (FISH) using a bacterial probe (orange), the e-cadherin (green) antibody, and DAPI (blue) stain (invasive bacteria identified by confocal microscopy Z-stacking are marked by white arrows) and **b** quantified using the gentamicin protection assay. **c** Activation of immune response was examined by qPCR of immune markers IL1β and IL6. LPS and AIEC were utilized as positive controls of immune activation and infection, respectively. Microscopy figures were imaged at × 63 magnification using a Leica SP5 confocal microscope. Statistical analysis was performed by two-tailed *t* test; *p < 0.05, **p < 0.01, ***p < 0.001, and ****p < 0.0001; NI: no infection control

Fluorescence microscopy (Fig. 5a) and gentamicin protection assay (Fig. 5b) were utilized as complementary approaches to examine the level of invasion of *B. cepacia* G143 25416, *Burkholderia vietnamiensis* DB01, *Burkholderia ambifaria* AMMD, *F. plautii* patient 94 (isolated from a CD patient), *Ruminococcus* sp*.* patient 94 (CD patient), *Ruminococcus* sp*.* patient 102 (non-IBD patient), and *Pseudomonas protogens*, along with adherent-invasive *E. coli* (AIEC; positive invasion control), *E. coli* HB101 (negative commensal control), and enterohemorrhagic *E. coli* (EHEC; negative invasion control), into HT29-MTX-E12 cells. Results from microscopy visually demonstrated invasion of *Burkholderia* species, *F. plautii* patient 94, *Ruminococcus* sp. patient 94, and *Ruminococcus* sp*.* patient 102 into intestinal epithelial cells in culture (Fig. 5a). To quantitatively assess this invasive capacity, we performed gentamicin protection assays, which showed that *B. cepacia* (*p* < 0.001), *B. ambifaria* AMMD (*p* < 0.001), *B. vietnamienesis* (*p* < 0.0001), *F. plautii* patient 94 (*p* < 0.001), and *Ruminococcus* sp*.* patient 94 (*p* < 0.0001) are found within the E12 epithelial cells following 24-h infection at a similar level compared to the invasive AIEC (*p* < 0.001; Fig. 5b). *Ruminococcus* sp. patient 102 only demonstrated low levels of invasion following 24-h infection of E12 epithelial cells (*p* = 0.015). As expected, EHEC and HB101 did not demonstrate any significant level of invasiveness. Interestingly, *P. protogens*, which displayed reduced ICI in IBD, also did not display statistically significant levels of invasion.

Furthermore, qPCR analysis demonstrated significant induction of the immune mediator, IL-6, in response to infection with *B. cepacia* (*p* < 0.05), *B. vietnamiensis* (*p* < 0.001), *B. ambifaria* (*p* < 0.05), *Ruminococcus* sp*.* patient 94 (*p* < 0.05), and *P. protogens* (*p* < 0.05; all relative to the negative control); this was comparable to the positive invasive control AIEC (*p* < 0.001) and LPS (*p* < 0.01; Fig. 5c). No significant change was found in IL-1β in any strain other than AIEC (*p* < 0.05). These findings support our hypothesis that binding to IgG is not random and is able to identify potential pathobionts, likely suggesting previous interaction of these specific bacteria with the host immune system.

## Discussion

The contribution of microbiota to the pathogenesis of IBD has been well established [52–54]; however, significant gaps remain in our understanding of the mechanisms involved and in the development of specifically tailored treatments directed towards microbial composition and function [55, 56]. In order to develop a better understanding of the etiology of IBD, we must first understand the complex interactions between the microbiota of the gut and the uncontrolled immune response of the host. However, methods currently available for use in identifying pathobionts, which may make up only a small percentage of the gut microbiota, remain challenging due to the high species diversity and complexity, location of potential pathobionts within the gut, interpersonal and intrapersonal variability, wide spectrum of IBD phenotypes, effects of treatments, and variable methodologies. Another potential issue with the design of previous studies is the predominant focus on the microbiota of the stool [24, 57], which differ from those found at the mucosa. Mucosa-associated microbes are more likely to be involved in disease pathogenesis due to their proximity to the intestinal epithelium and the underlying immune system [58]. These issues were addressed in the current study by isolating large quantities of mucosa-associated microbes through intestinal washes combined with high throughput cell sorting techniques. This allowed us to focus on the microbes most likely to induce an immune response at the location they are most likely to be found.

The immune system of the human intestinal mucosa plays a critical role in maintaining a homeostatic environment by selectively identifying pathogenic microorganisms while aiding in the sustained growth of harmless/beneficial commensal microorganisms [28, 59]. Majority of immune receptors interact with bacteria via components such as lipopolysaccharides, which are present in both pathogenic and commensal bacteria [59]; therefore, the precise mechanism implemented by the immune system to differentiate pathogens from commensals is complex and requires both innate and adaptive responses. The immune system is thought to identify pathogens by detecting bacterial adhesive or invasive activities through various cell and receptor interactions [60, 61].

Studies have demonstrated that pathobionts are capable of penetrating both mucus and epithelial layers of the intestine, thereby interacting with, and activating, the immune system [62]. One important characteristic of the intestinal adaptive immune response is the production of large amounts of IgA or IgG antibodies, produced in response to invasive microorganisms [61]. We therefore hypothesized that the host IgG immune response, whereby IgG naturally binds to select gut microbes within the patient gut, could be used as a marker to identify bacteria more likely to be virulent and potentially involved in IBD pathogenesis.

Coating of bacteria with Ig has previously been investigated in IBD mouse models and in human patients [32, 63], although live culture or sequencing experiments have rarely been involved in these studies. While most of these studies have focused on IgA-bound bacteria, for the reasons highlighted in the “Introduction” section, we were interested in those naturally bound by IgG. Importantly, in attempts to better understand the origins of IBD, the focus of this study was on children with IBD as pediatric patients display more recent disease onset, relative lack of co-morbidities, more extensive phenotype [64], and reduced exposure to environmental confounders (e.g., smoking) [65, 66]. In order to examine the intestinal immune response to the gut microbiota of pediatric IBD patients, we collected mucosa-associated bacteria during endoscopy and then by IgG-based cell sorting identified taxa bound to IgG using Illumina MiSeq sequencing of 16S rRNA gene and shotgun metagenomics libraries. A similar method has been used previously by our team and others [28, 67], and optimization of next-generation sequencing techniques has identified the use of 16S rRNA gene and shotgun metagenomic libraries as superior approaches for combining taxonomic identification with functional information for the study of the intestinal microbiome [67].

Little is known about the precise role of IgG binding of bacteria in IBD compared to normal controls. Unlike secretory IgA, it is unclear whether IgG is involved in the immune tolerance of intestinal microbiota as it is generally secreted in response to invading pathogens able to penetrate through the mucosal barrier. Recent evidence suggests, that under homeostatic conditions, symbiotic microbes can disseminate systemically, inducing an IgG response [68], primarily targeting Gram-negative bacteria. Our analysis indicates an increase in IgG binding by Firmicutes and Proteobacteria, which are Gram-positive and Gram-negative, respectively, suggesting the potential for pathobionts disseminating and being detected outside of the lumen. CD patients have higher IgG-bound bacteria in stool, which has been suggested as a loss of mucosal tolerance [69]. However, our data suggests that these bacteria, recognized as more highly bound by IgG, may be pathobionts as the in vitro experiments show them to have increased invasion potential. These pathobionts likely play a role in promoting inflammation and/or altering the gut microenvironment to improve pathogenicity.

Here, we demonstrate that the binding of IgG favors different subsets of specific microbes in non-IBD, CD, or UC. The original study design aimed to assess multiple patient profiles using 16S rRNA gene approaches. These results offered only a limited taxonomical and functional resolution; therefore, the focus of the study was supplemented by shotgun metagenomics. The small number of patients examined proved to be a limitation in this study, especially for the shotgun metagenomics analysis (*n* = 9 in total; this was due to low quality and quantity DNA after FACS of luminal samples, leading to loss of DNA); however, this can be overcome in future studies designed to examine specific changes in microbiota while the current study provides proof of principle for the use of IgG as a marker of pathogenicity.

Interestingly, a number of species were found to display increased or decreased ICI within our patient cohort, which was found to be unrelated to any changes in species abundance within these samples (Figs. 2c and 3c, Additional file 1: Figures S2 and S3). Although there was great interpersonal-variability between the pediatric patients of each cohort, we found that the mucosa-associated bacteria that we identified with greater amounts of IgG binding included select species, some of which have known pathogenic potential or have been previously linked to inflammation, while others have not and are potential novel pathobionts. Use of IgG as a marker allowed for the identification of specific bacterial species, one of which, *Burkholderia cepacia*, has previously been identified in ileal biopsies taken from pediatric IBD patients [45]. We, therefore, chose to focus on *B. cepacia* to support our hypothesis and have demonstrated increased IgG binding of *B. cepacia* in UC patients compared to non-IBD patients. To further support these claims, we also examined the anaerobic bacteria *F. plautii* and *Ruminococcus* sp., which display increased ICI in CD patients compared to non-IBD patients and were isolated live from our pediatric cohort. When reintroduced into human intestinal cell cultures, these bacteria, which were more highly bound by IgG, demonstrated invasive capacity and induced a pro-inflammatory immune response; thus, our data suggest that IgG coating selectively labels inflammatory microorganisms potentially involved in IBD pathogenesis. Interestingly, we were able to isolate *Ruminococcus* sp. from both CD (pt 94) and non-IBD (pt 102) patients and results indicated that the species isolated from CD displayed greater invasive properties, indicating that differences in the microenvironment of the IBD gut may play a role in shaping the invasiveness of these microorganisms. We further supported the use of IgG as a marker of pathobionts in IBD by examining *P. protogens*, which displayed statistical significant reduction in ICI. *P. protogens* was not significantly invasive in vitro and elicited a minimal IL-6 response following infection.

## Conclusions

Here, we verified that increased ICI depicts increased invasive capacity, demonstrating in proof of principle that our method of using ICI to identify bacteria that may be important in establishing and/or exacerbating the inflammation in IBD patients is valid. Larger studies are certainly required to confirm our findings and direct further research into novel pathobionts. Elucidating the role of specific bacterial species in IBD pathogenesis will underpin new strategies to improve our ability to direct therapies to those patients most likely to respond by utilizing personalized therapies targeted towards replacement or exclusion of those intestinal microbes involved in IBD development and progression. Furthermore, this technique has implications beyond the scope of the present study as alterations in the gut microbiota have been connected to a number of diseases from autism to cancer [15–23]. Utilizing the techniques validated in this study to identify potential pathobionts, or microbes recognized by the patient IgG, in other disease settings could prove to be a powerful tool in tailored therapeutic options for a large number of patients.

## Materials and methods

### Consent and ethics approval

Consent was obtained from patients/guardians, and the study was approved by the Health Research Ethics Board at the University of Alberta (Study ID Pro00023820), Edmonton, AB, Canada.

### Patient criteria and sample collection

Patients aged 3–18 years, with histological and endoscopic confirmed diagnosis of CD or UC, based on the revised Porto criteria [70] and the Paris classifications [66] were eligible to participate; non-IBD controls underwent colonoscopy for abdominal pain and/or diarrhea, but endoscopy and histology were completely normal, excluding the possibility of IBD. Detailed inclusion and exclusion criteria were described previously [27, 71]. UC subjects endoscopically or histologically diagnosed with backwash ileitis were excluded from the study. For all patients, bowel cleansing was standardized using Picosalax® (sodium picosulfate and magnesium oxide) prior to endoscopy. Aspirate washes from the TI were collected from patients during endoscopy, and before biopsies were obtained at the Stollery Children’s Hospital, University of Alberta in Edmonton, Alberta, Canada. Protease inhibitor (1% *v*:*v*; Sigma Aldrich) was added immediately to the aspirate to prevent degradation and further purified by filtration (40-μm filters) and centrifugation at 200 g (5 min, 4 °C) to discard food particles and human cells. The supernatant-containing bacteria were washed thrice in PBS (14,000 g, 5 min, 4 °C) and fixed in 4% (*v*:*v*) paraformaldehyde for 1 h, then washed and stored in 0.2% (*w*:*v*) BSA/PBS.

### Immunofluorescence of cytometry samples

All buffers and solutions used were filter-sterilized, and samples were washed in 0.2% (*v*:*v*) Tween-20 in PBS, including two centrifugation steps (3700 g, 10 min, 4 °C). Samples were blocked (5% *w*:*v* BSA/2% *v*:*v* goat serum) for 30 min, washed, and then aliquoted for staining.

For FACS Aria cell sorting and ImageStream image cytometry, samples were aliquoted to 3 groups: an unstained control, an IgG isotype control (Abcam; diluted 1:100; 2 μg/mL), and an anti-human IgG stained sample (Abcam; diluted 1:150; 3 μg/mL). Aliquots were washed and incubated with AlexaFluor 488-conjugated anti-rabbit secondary antibody (Invitrogen; diluted 1:300; 7 μg/mL). To identify live bacterial cell populations, aliquots containing IgG isotype and IgG were stained with either propidium iodide (PI; Life Technologies; diluted 1:5; 50 μg/mL) 15 min prior to sorting FACS Aria samples, or with 4′,6-diamidino-2-phenylindole (DAPI; Thermo Fisher; diluted 1:1000; 1 μg/mL) immediately prior to image flow cytometry.

### Fluorescence-activated cell sorting (FACS)

The sensitivity of FACS to sort fluorescent bacteria from non-fluorescent bacteria was determined in initial setup of the protocol using *E. coli* HB101 (Additional file 1: Figure S1). *E. coli* HB101 were seeded at 1 × 10^6^ (CFU/mL) and stained using a polyclonal rabbit anti-*E. coli* O&K lipopolysaccharide (LPS) antibody (Invitrogen; diluted 1:100; 2 μg/mL), washed and secondary stained with an FITC anti-rabbit secondary antibody (Invitrogen; diluted 1:400; 5 μg/mL). Flow cytometry was utilized to detect LPS surface staining. Fluorescence-activated cell sorting (FACS) analysis was performed on a co-culture of *E. coli*, and *L. reuteri* stained with an anti-*E. coli* LPS Ab. *E. coli* segregated by FACS were grown on MacConkey and MRS agar plates. The purity of both *E. coli* and *L. reuteri* samples separated by FACS was confirmed by qPCR. DNA extraction was performed using a QIAGEN DNeasy mini spin column kit, and subsequent real-time PCR was performed using primers targeted to *E. coli* (FWD: 5′CATGCCGCGTGTATGAAGAA; RVS: 5′CGGGTAAACGTCAATGAGCAAA), and *L. reuteri* (FWD: 5′AGCAGTAGGGAATCTTCCA; RVS: 5′CACCGCTACACATGGAG).

For fixed cell sorting of patient wash samples, the cell concentration was set at 25,000 events/s with a 70-μm nozzle (70 psi). The quadrants comprising of PI positive/IgG negative (PI+IgG−) and PI positive/IgG positive (PI+IgG+) were sorted from a quadrant plot and collected in 15-ml conical tubes (1 × 10^7^ events per tube). Data were collected using a BD FACSAria III cytometer (BD Bioscience) equipped with the appropriate lasers. The sorted cells were centrifuged, and the pellet was frozen at − 80 °C for 16S rRNA gene or shotgun metagenomic analyses. Data files were analyzed using FlowJo V7 software (FlowJo LLC).

### Image cytometry

In parallel to flow cytometry, a cohort of mucosal washings were stained and assessed by image cytometry to demonstrate the specificity of IgG antibody binding for the purpose of accurate cell sorting. A 60-μL aliquot was taken from a portion of patient samples prior to staining for FACS Aria. Samples were stained using the universal FISH probe EUB338 (diluted 1:100; 0.5 μg/mL; 5′G*CTGCCTCCCGTAGGAGT-3′[Cy3]) [72]. Hybridization of bacterial cells in suspension was carried out in a water bath for 2 h at 50 °C (900 mM NaCl, 20 mM Tris–HCl pH 7.4, 0.05% SDS [*w*:*v*]). Vials were washed for 5 min twice at 50 °C (20 mM Tris–HCl pH 7.4, 0.006% SDS [*w*:*v*]) and then blocked for 30 min (5% [*v*:*v*] goat serum and 2% [*w*:*v*] bovine serum albumin). Samples were then stained using the IgG (Abcam; diluted 1:100; 3 μg/mL) antibody followed by AlexaFluor 488-conjugated anti-rabbit secondary (Life Technologies; diluted 1:400; 7 μg/mL). Vials were stringently washed between steps thrice with 0.05% (*v*:*v*) Tween-20. To determine the presence of bacterial DNA, vials were counterstained with DAPI (diluted 1:1000; 1 μg/mL) for 1 min.

The Amnis ImagestreamX mkII was used for acquisition performed at × 60 magnification on a low-speed setting with a total of 100,000 events collected. For gating compensation, each run included controls stained only with DAPI, EUB338, or anti-IgG. Samples were gated on selecting singlet events of DNA-positive bacteria. IDEAS Software version 6.1 (Amnis) was employed for analysis as previously described [73].

### NGS libraries construction and bioinformatics

#### 16S rRNA gene library

DNA extraction was performed using the QIAGEN DNeasy mini spin column kit. Library construction, sequencing, and bioinformatics analysis were conducted as described in Alipour et al., 2016 [27, 74, 75]. In brief, the cDNA created from bacterial isolates of intestinal washes was used as template DNA using primers targeted towards the V3 and V4 variable regions of the 16S rRNA gene in a nested PCR [Forward 5′-TCGTCGGCAGCGTCAGATGTGTATAAGAGACAGC CTACGGGNGGCWGCAG-3′;Reverse 5′-GTCTCGTGGGCTCGGAGATGTGTATAAGAGACAGGACTAC HVGGGTATCTAATCC-3′]. A 25-cycle PCR was run (denature 95 °C [30 s], anneal 55 °C [30 s], elongation 72 °C [30 s]), and PCR amplicons were purified using AMPure XP paramagnetic beads [Beckman Coulter, Brea, CA]. For profiling of bacteria in total aspirate washes, or in fractions thereof that did or did not bind anti-IgG, the V3–V4 regions of the 16S ribosomal RNA gene were PCR amplified and sequenced on a MiSeq instrument (Illumina), according to procedures described in the 16S Sample Preparation Guide 15044223 A (Illumina, San Diego, CA). In brief, ~ 1 ng of microbial DNA was used for a gene-specific PCR with primers containing an Illumina linker at its 5′ moiety for 25 cycles. This produced an amplicon of approximately 550 bp flanked by the Illumina linkers. Subsequently, an indexing PCR was conducted for 18 cycles using primers flanked by Illumina NexteraXT adapters, suitable for binding to oligos on the flow-cell of the MiSeq instrument. Libraries were then cleaned up with AMPure paramagnetic beads, and quantified using a protocol that combines the average size of the library as measured in a 2100 Agilent Bioanalyzer instrument and the dsDNA concentration as determined by Qubit. Sequencing was conducted using a demultiplexing and adapter trimming protocol with a 600 cycles V3 sequencing kit (Illumina).

For bioinformatics analysis, sequences were inspected and per-base quality scores determined. Sequencing primers were removed and additional bases at the 5′ and 3′ ends of each sequence were trimmed off when quality scores (Q) were < 20. Sequences were further processed using the QIIME pipeline. Sequences were clustered into OTUs using UCLUST, chimeric sequences were removed with USEARCH, and taxonomy assignments done with the RDP classifier [67].

#### Shotgun metagenomics

Shotgun libraries were constructed using the Nextera XT® kit (Illumina) according to manufacturer’s instruction. Five microliters of 0.2 ng/μl microbial genomic DNA was supplemented with 10 μl of TD buffer and 5 μl of ATM buffer, for tagmentation at 55 °C for 5 min. The reaction was neutralized by adding 5 μl of NT buffer and then incubated for 5 min at RT. Tagmented DNA was supplemented with 15 μl of Nextera PCR master mix (NPM) and 5 μl of each indexing oligo and then subjected to PCR amplification for 12 cycles. Libraries were cleaned up, quantified, and sequenced as described for 16S rRNA gene libraries. Sequencing adapters trimming and demultiplexing of libraries were conducted in-instrument. Sequences were taxonomically classified with KraKEN [45, 74] using a customized database containing full-genome sequences of bacteria, archaea, viruses, fungi, protozoa (refseq database from NCBI), and the human genome assembly GRCh38. Sequences abundance was re-estimated using Bracken (Bayesian Reestimation of Abundance after Classification with KrakEN) [75].

### Bacterial strains and cell line culturing conditions

The HT-MTX29-E12 cell line was obtained from American Type Culture Collection. E12 cells were maintained in RPMI supplemented with Fetal bovine serum (10%, *v*:*v*) and penicillin/streptomycin (Gibco; 100 μg/mL) at 37 °C and 5% CO_2_.

Bacterial strains *Burkholderia cepacia* G143 25416, *Burkholderia vietnamiensis* DB01, and *Burkholderia ambifaria* AMMD were generously provided by Dr. Jonathan Dennis (University of Alberta). The strains were grown on 50% Luria-Bertani growth medium (12.5 g/L; BD biosciences) at 37 °C.

*Pseudomonas protogens* was purchased from the ATCC and was cultured at 30–37 °C in LB media.

*Escherichia coli* HB101, enterohemorrhagic *E. coli* (EHEC) O157:H7, and adhesive invasive *E. coli* (AIEC) LF82 were cultured in LB at 37 °C.

*Lactobacillus reuteri* was cultured in MRS medium at 37 °C. All strains above were grown under aerobic conditions.

Bacterial strains *Flavonifractor plautii* patient 94, *Rumminococcus* sp*.* patient 94, and *Rumminococcus* sp*.* patient 102 were isolated live from a patient with Crohn disease (patient 94) or non-IBD (patient 102) by growing cultures for individual colonies on BHI plates under anaerobic conditions. Individual colonies were isolated and identified by 16S rRNA gene sequencing.

### Bacterial infection

Bacterial isolates were grown on LB agar plates at room 37 °C. When visible growth was observed, a single colony was grown in LB liquid medium overnight. An OD_600_ of 0.1 (approx. 1 × 10^8^ cfu/mL) was inoculated to 10 mL Dulbecco modified Eagle medium (DMEM) (Life Technologies) supplemented with 15 mM Hepes (Gibco) and 1% (*v*:*v*) non-essential amino acid (NEAA) supplement (Gibco) and grown overnight prior to infection of human epithelial cells. Human epithelial cell cultures were inoculated with bacteria (roughly 120:1 multiplicity of infection) or left uninfected. Infection was carried out at 37 °C and 5% CO_2_ for 24 hs.

### Fluorescent in situ hybridization (FISH)

FISH was performed on the E12 human intestinal epithelial cell line seeded on 12-mm glass coverslips, post-infection, using the universal FISH probe EUB338 (diluted 1:100; 0.5 μg/mL). For aerobe cultures, bacteria were pre-stained with EUB338 for 2 h prior to infection to reduce background staining. For anaerobes, staining with EUB338 was performed following infection.

Following infection, the non-adhered bacteria were aspirated and replaced with growth medium containing 100 μg/mL of gentamicin for 2 h to inhibit extracellular overgrowth (all bacterial strains were confirmed to be sensitive to gentamicin). Supernatants were then aspirated and cells were washed in PBS, fixed, and permeabilized using 4% (*v*:*v*) paraformaldehyde for 20 min at room temperature. Coverslips were stained using the common primer probe EUB338 (diluted 1:100; 0.5 μg/mL) through the process of hybridization (buffer: 0.02 M Tris–HCl pH 7.4, 0.9 M NaCl, 5% [*v*:*v*] formamide, 0.05% [*w*:*v*] SDS) at 40 °C for 2 h and then washed (buffer: 0.02 M Tris–HCl pH 7.4, 0.66 M NaCl, 0.006% [*w*:*v*] SDS) at 40 °C for 10 min. To visualize human epithelial cells, coverslips were blocked (buffer 2% [*v*:*v*] goat serum, 1% [*w*:*v*] BSA) for 30 min and stained by immunofluorescence using Philloidin 488 antibody (diluted 1:40; 6 μg/mL in 0.2% [*v*:*v*] goat serum, 0.1% [*w*:*v*] BSA; BD Bioscience) for 1 h, followed by 1 min DAPI stain to highlight bacteria and epithelial cell nuclei (diluted 1:1000; 1 μg/mL). Mounted coverslips were visualized on the Zeiss Fluorescence microscopy system after allowing fluorescent stain to develop for 24 h.

### Gentamicin protection assay

Following infection of the human intestinal epithelial cell line HT29-MTX-E12, non-adhered bacteria were aspirated and replaced with growth medium containing 100 μg/mL of gentamicin for 2 h to kill all remaining extracellular bacteria (gentamicin is not lipophilic and therefore, does not penetrate the eukaryotic cell membrane; as a result, invasive bacteria were unaffected. Invasive bacteria were collected by lysing human cells with 50% (*v*:*v*) TritonX-200 for 5 min at room temperature, followed by serial dilutions and plating on LB ampicillin (50 μg/mL) agar plates, grown overnight at 37 °C, to quantify bacterial invasion by colony forming units by manual counting.

### qPCR for cytokine response to infection

RNA was isolated from E-12 cells as previously described [76] following 24 h infection with AIEC, *B. cepacia*, *B. vietnamiensis*, or *B. ambifaria* as indicated. LPS (10 nM) was utilized as a positive control for immune activation. RT-PCR was performed as previously described [76]. Both biological and technical replicates were performed on all reaction using primers for the following genes: GAPDH, IL-1β, and IL-6 (Table 2). Data were analyzed using CFX Manager Software Version 3.0 (Bio-Rad Laboratories, Inc.).

**Table 2:** Primer sequences

Primer target	FWD sequence	REV sequence
GAPDH	5′-CCC ACT CCT CCA CCT TTG AC-3′	5′-ATG AGG TCC ACC ACC CTG TT-3′
IL1β	5′-TCC GAC CAC CAC TAC AGC AA-3′	5′-ATC TTT CAA CAC GCA GGA CA-3′
IL6	5′-CCA CAC AGA CAG CCA CTC AC-3′	5′-AGG TTG TTT TCT GCC AGT GC-3′

### Data analysis

Results were expressed as mean ± standard error of mean (SEM). The statistical analyses were performed using InStat 3.0 (GraphPad Software, San Diego, CA, USA). Comparisons between the means of 3 or more groups were made with the non-parametric Kruskal-Wallis analysis of variance (ANOVA) and Dunn’s multiple comparison tests. Significance: one asterisk, *p* < 0.05; two asterisks, *p* < 0.01; and three asterisks *p* < 0.001.

Statistical significance of qPCR was evaluated by unpaired *t* test with Welch’s correction using GraphPad Prism 4.0 (GraphPad Software).

## Additional file

Additional file 1: **Table S1.** Patient characteristics. **Table S2.** Detailed patient characteristics and diagnosis. **Table S3.** Detailed average quadrant 1 and quadrant 2 percentages and standard deviations of FACS sorting for pediatric IBD patient wash samples. **Figure S1.** Validation of flow cytometry and cell sorting for bacterial isolation. **Figure S2.** ICI scores of species identified in the shotgun metagenomics library in non-IBD and IBD. **Figure S3.** ICI scores of species identified in the shotgun metagenomics library of CD or UC. (ZIP 1310 kb)

## Abbreviations

BSA: Bovine serum albumin
CD: Crohn disease
DAPI: 4′,6-Diamidino-2-phenylindole
FACS: Fluorescence-activated cell sorting
FISH: Fluorescence in situ hybridization
IBD: Inflammatory bowel diseases
IgG: Immunoglobulin G
IL: Interleukin
PBS: Phosphate-buffered saline
TI: Terminal ileum
UC: Ulcerative colitis
