# Supplementary material for: Host immunoglobulin G selectively identifies pathobionts in pediatric inflammatory bowel diseases
Source: Microbiome. 2019 Jan 3;7:1. doi: 10.1186/s40168-018-0604-3 (PMC6317230; doi:10.1186/s40168-018-0604-3)
Supplement: Supplementary file 1 — Table S1. Patient characteristics. Table S2. Detailed patient characteristics and diagnosis. Table S3. Detailed average quadrant 1 and quadrant 2 percentages and standard deviations of FACS sorting for pediatric IBD patient wash samples. Figure S1. Validation of flow cytometry and cell sorting for bacterial isolation. Figure S2. ICI scores of species identified in the shotgun metagenomics library in non-IBD and IBD. Figure S3. ICI scores of species identified in the shotgun metagenomics library of CD or UC. (ZIP 1310 kb) [file 40168_2018_604_MOESM1_ESM.zip › IgG Microbiome Oct 12 2018 Suppl tables and fig legands.docx]

**Additional file:**

**Table S1.** Patient characteristics.

| **Patient Characteristic** | **Non-IBD** | **CD** | **UC** |
| --- | --- | --- | --- |
| *N* | 10 | 15 | 7 |
| Mean age, years (range) | 13.4 (7-17) | 12.6 (3-17) | 12.7 (6-15) |
| Gender (F/M) | 8/2 | 6/9 | 6/3 |
| Disease activity ^a^ | NA | 2/7/4/2 | 4/1/4/0 |
| Diagnosis (new/follow up) | NA | 6/9 | 2/7 |
| Treatments at time of endoscopy (%) |  |  |  |
| Azathioprine | 0 | 13 | 44 |
| Infliximab | 0 | 33 | 11 |
| Prednisone | 0 | 0 | 11 |
| 5-aminosalicylic acid | 0 | 6 | 44 |
| Methotrexate  Proton pump inhibitor | 0  10 | 27  0 | 11  0 |

^a^ Disease activity (remission/mild/moderate/severe) was based on Paediatric Crohn’s Disease Activity Index (PCDAI) and Paediatric Ulcerative Colitis Activity Index (PUCAI). NA, not applicable; F, female; M, male.

**Table S2:** Detailed patient characteristics and diagnosis.

**Table S3:** Detailed average quadrant 1 and quadrant 2 percentages and standard deviations of FACS sorting for pediatric IBD patient wash samples.

| Type | Sort % | | STDEV | |
| --- | --- | --- | --- | --- |
|  | PI+IgG- | PI+IgG+ | PI+IgG- | PI+IgG+ |
| Non-IBD | 34.5 | 33.1 | 14.4 | 11.5 |
| CD | 24.2 | 28.1 | 14.2 | 11.4 |
| UC | 37.1 | 36.0 | 13.1 | 9.8 |

**Supplementary Figure Legends:**

**Figure S1: (A) *E. coli* are positively identified by flow cytometry using an anti-*E. coli* LPS antibody.** 1×10^6^ *E. coli* were incubated with a polyclonal rabbit-anti *E. coli* O&K LPS Ab. LPS surface staining was subsequently determined by flow cytometry**. (B) Fluorescence-activated cell sorting (FACS) effectively isolates *E. coli* from a mixed-culture of *E. coli* and *Lactobacillus reuteri*.** *E. coli* and *L. reuteri* were co-cultured and stained with an anti-*E. coli* LPS Ab. ~1×10^6^ *E. coli* were separated out by FACS gaiting and sorted for *E. coli* Ab-positive bacteria. Purity and viability were determined by growth patterns on specific agar plates (both MacConkey and MRS). **(C)** Specificity of FACS isolation of *E. coli* was confirmed by RT-qPCR performed on bacterial isolates from *E. coli* O&K positive and *E. coli* O&K negative samples.

**Figure S2:** ICI scores of species identified in the shotgun metagenomics library in non-IBD and IBD. Ratio of IgG binding (ICI score) to bacterial species of interest in the shotgun metagenomics library patient samples where each dot represents an individual patient. ICI for individual species is segregated based on preferential binding to that species in **(A)** non-IBD, **(B)** neither non-IBD or IBD, or **(C)** both CD and UC. Statistical analysis was performed by ANOVA.

**Figure S3:** ICI scores of species identified in the shotgun metagenomics library of CD or UC. Ratio of IgG binding (ICI score) to bacterial species of interest in the shotgun metagenomics library patient samples where each dot represents an individual patient. ICI for individual species is segregated based on preferential binding to that species in **(A)** CD, or **(B)** UC. Statistical analysis was performed by ANOVA.
