# Supplementary figures and images for: Host immunoglobulin G selectively identifies pathobionts in pediatric inflammatory bowel diseases
Source: Microbiome. 2019 Jan 3;7:1. doi: 10.1186/s40168-018-0604-3 (PMC6317230; doi:10.1186/s40168-018-0604-3)

## Slide 1
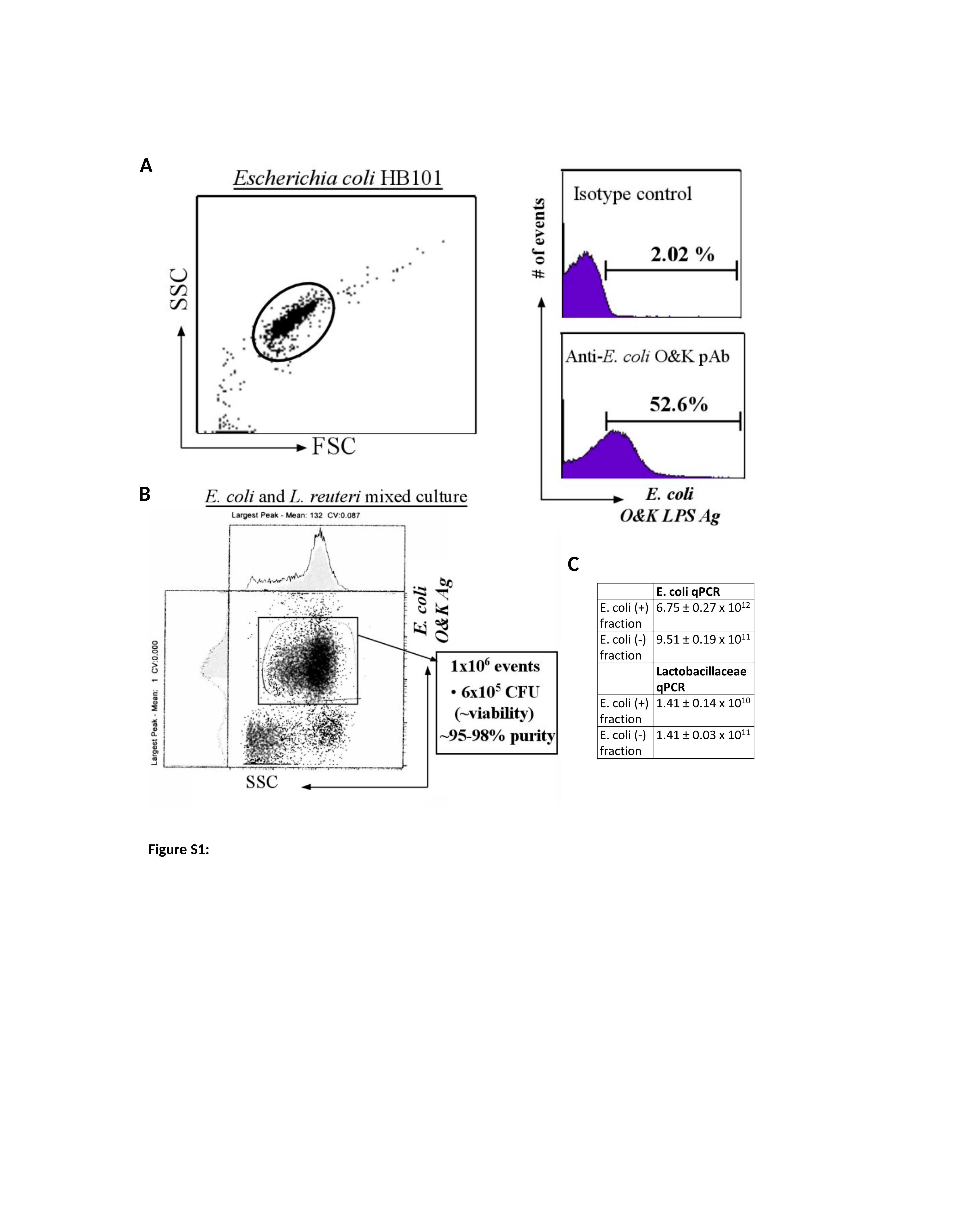

A
B
C
Figure S1:

Supplement: Supplementary file 1 — Table S1. Patient characteristics. Table S2. Detailed patient characteristics and diagnosis. Table S3. Detailed average quadrant 1 and quadrant 2 percentages and standard deviations of FACS sorting for pediatric IBD patient wash samples. Figure S1. Validation of flow cytometry and cell sorting for bacterial isolation. Figure S2. ICI scores of species identified in the shotgun metagenomics library in non-IBD and IBD. Figure S3. ICI scores of species identified in the shotgun metagenomics library of CD or UC. (ZIP 1310 kb) [file 40168_2018_604_MOESM1_ESM.zip › IgG Microbiome Oct 12 2018 FIG S1.pptx]

## Slide 1
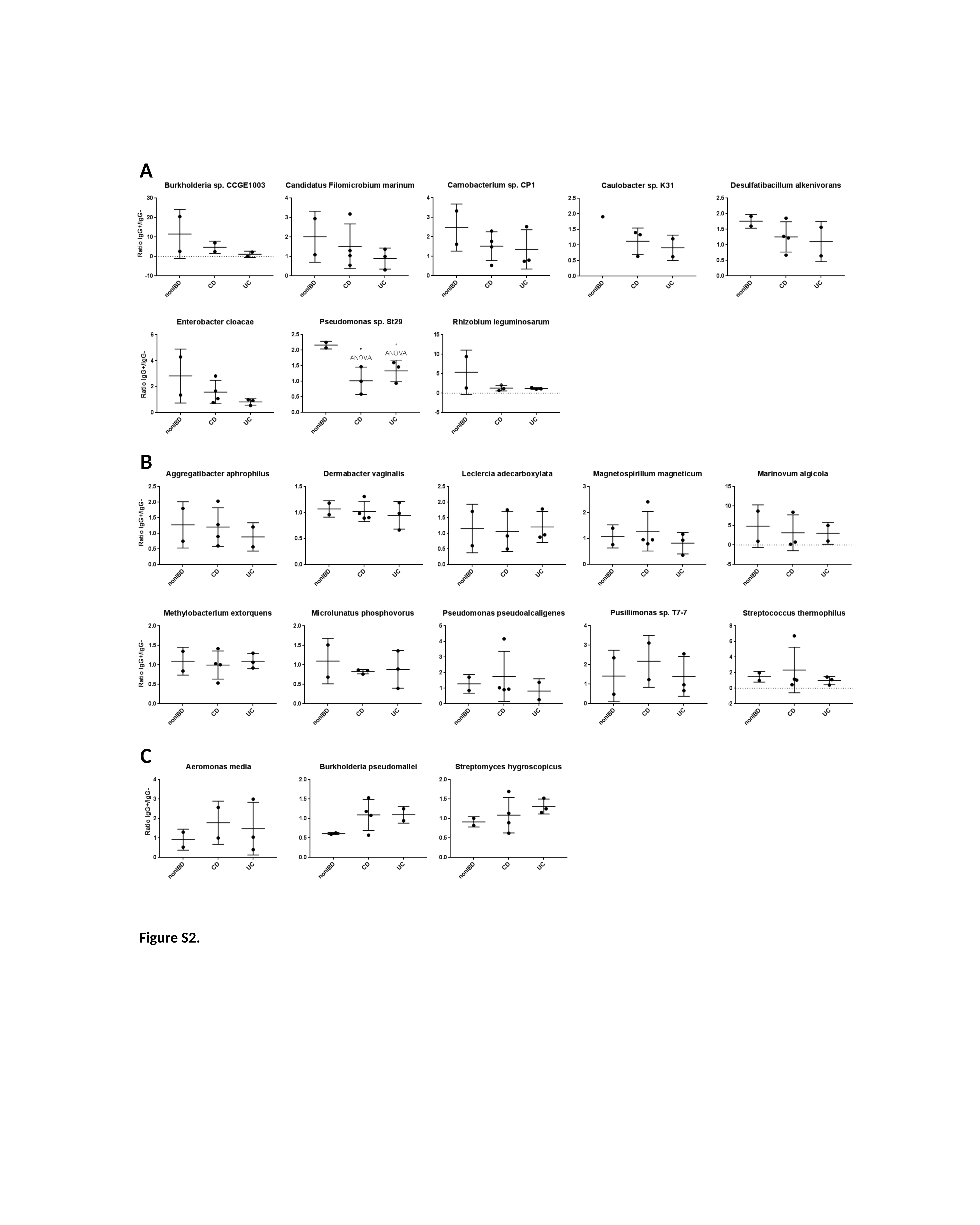

A
B
C
Figure S2.

Supplement: Supplementary file 1 — Table S1. Patient characteristics. Table S2. Detailed patient characteristics and diagnosis. Table S3. Detailed average quadrant 1 and quadrant 2 percentages and standard deviations of FACS sorting for pediatric IBD patient wash samples. Figure S1. Validation of flow cytometry and cell sorting for bacterial isolation. Figure S2. ICI scores of species identified in the shotgun metagenomics library in non-IBD and IBD. Figure S3. ICI scores of species identified in the shotgun metagenomics library of CD or UC. (ZIP 1310 kb) [file 40168_2018_604_MOESM1_ESM.zip › IgG Microbiome Oct 12 2018 FIG S2.pptx]

## Slide 1
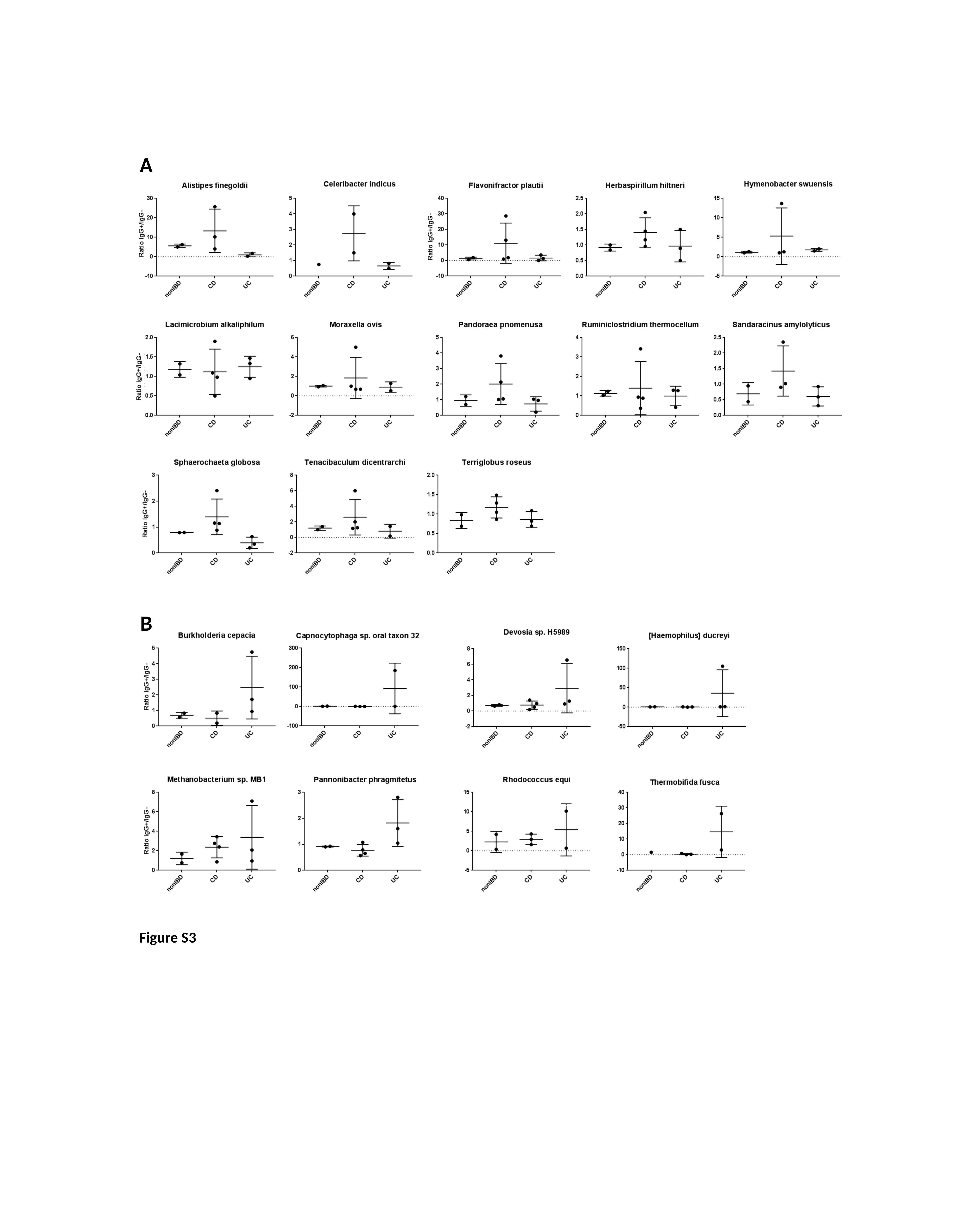

A
B
Figure S3

Supplement: Supplementary file 1 — Table S1. Patient characteristics. Table S2. Detailed patient characteristics and diagnosis. Table S3. Detailed average quadrant 1 and quadrant 2 percentages and standard deviations of FACS sorting for pediatric IBD patient wash samples. Figure S1. Validation of flow cytometry and cell sorting for bacterial isolation. Figure S2. ICI scores of species identified in the shotgun metagenomics library in non-IBD and IBD. Figure S3. ICI scores of species identified in the shotgun metagenomics library of CD or UC. (ZIP 1310 kb) [file 40168_2018_604_MOESM1_ESM.zip › IgG Microbiome Oct 12 2018 FIG S3.pptx]
